# Potential application of phage vB_EfKS5 to control *Enterococcus faecalis* and its biofilm in food

**DOI:** 10.1186/s13568-023-01628-6

**Published:** 2023-11-20

**Authors:** Mohamed El-Telbany, Chen-Yu Lin, Marwa Nabil Abdelaziz, Aye Thida Maung, Ayman El-Shibiny, Tahir Noor Mohammadi, Mahmoud Zayda, Chen Wang, Su Zar Chi Lwin, Junxin Zhao, Yoshimitsu Masuda, Ken-ichi Honjoh, Takahisa Miyamoto, Mohamed El

**Affiliations:** 1 Department of Bioscience and Biotechnology, Graduate School of Bioresource and Bioenvironmental Sciences, Kyushu University, Fukuoka, Japan; 2 Department of Microbiology and Botany, Faculty of Science, Zagazig University, 44519 Zagazig, Egypt; 3 Center for Microbiology and Phage Therapy, Zewail City of Science and Technology, 12578 Giza, Egypt; 4 Teagasc Food Research Center, Moorepark, Fermoy, Cork Ireland; 5 Department of Food Hygiene and Control, Faculty of Veterinary Medicine, University of Sadat City, Sadat City, Monofiya Governorate Egypt; 6 Department of Bioscience and Biotechnology, Faculty of Agriculture, Graduate School, Kyushu University, 744 Motooka, Nishi-ku, 819-0395 Fukuoka, Japan

**Keywords:** *Enterococcus faecalis*, Bacteriophages, Biofilm, Nisin, Food application

## Abstract

Contaminated food with antibiotic-resistant *Enterococcus* spp. could be the vehicle for transmitting *Enterococcus* to humans and accordingly cause a public health problem. The accumulation of biogenic amines produced by *Enterococcus faecalis* (*E. faecalis*) in food may have cytological effects. Bacteriophages (phage in short) are natural antimicrobial agents and can be used alone or in combination with other food preservatives to reduce food microbial contaminants. The aim of this study was to isolate a novel phage against *E. faecalis* and determine its host range to evaluate its potential application. Bacteriophage, vB_EfKS5, with a broad host range, was isolated to control the growth of *E. faecalis*. The vB_EfKS5 genome is 59,246 bp in length and has a GC content of 39.7%. The computational analysis of phage vB_EfKS5 genome confirmed that it does not contain any lysogenic, toxic, or virulent genes. Phage vB_EfKS5 exhibited lytic activity against most *E. faecalis* isolates with different multiplicities of infections and it infected 75.5% (22/29) of *E. faecalis* isolates and 42.3% (3/7) of *E. faecium* isolates. It was also able to destroy the biofilm formed by *E. faecalis* with different MOIs. Phage vB_EfKS5 alone or in combination with nisin could control the growth of *E. faecalis* in broth and milk. Based on its high productivity, stability, short latent period, and large burst size, phage vB_EfKS5 has a high potential for applications both in food and medical applications.

**Supplementary Information:**

The online version contains supplementary material available at 10.1186/s13568-023-01628-6.

## Key points

A total of 28 *E. faecalis* isolates were isolated from different food samples.

Phage vB_EfKS5 was successfully characterized and found to inhibit the growth of *E. faecalis* as well as destroy its biofilms.

Phage-nisin combination exhibited a synergistic effect in eliminating *E. faecalis*.

**Supplementary Information:**

The online version contains supplementary material available at 10.1186/s13568-023-01628-6.

## Introduction

Members of *Enterococcus* spp. are versatile organisms that are recognized as opportunistic nosocomial pathogens, food spoilage bacteria, and starter cultures in fermented food (Giraffa 2003). Despite being found among normal microbiota that play an important role in the ripening of traditional cheese varieties, *E. faecalis* is regarded as a major contaminant that withstands harsh environmental conditions and persists in dairy equipment and facilities (Linares et al. 2016). Furthermore, *E. faecalis* can harbor antimicrobial resistance genes (AMR) that can be transferred by horizontal gene transfer to the surrounding microbiota. The contamination of food by *E. faecalis* can be achieved via several sources such as contaminated milk and/or water or by cross-contamination during cheese manufacturing **(**Linares et al. 2012). The production of proteolytic and lipolytic enzymes by these bacteria leads to changes in the taste and flavor of food. Moreover, *E. faecalis* could produce Biogenic Amines (BA_S_) tyramine and putrescine that may be cytotoxic and genotoxic for intestinal cells **(**Linares et al. 2016). The consumption of food containing high levels of these compounds can lead to several serious diseases such as headaches, migraines, and hypertension that can affect healthy and immunocompromised patients **(**del Rio et al. 2017; 2019). In addition, the biofilms and genome plasticity of these bacteria are key factors in their distinguished ability to acquire and trade antimicrobial resistance genes (Torres et al. 2018). The resistance of *E. faecalis* to several antibiotic classes enhances their ability to cause several infections **(**Cui et al. 2020). Additionally, *E. faecalis* virulence genes enhanced its ability to form strong biofilms **(**Sarantinopoulos et al. 2006). Virulence factors included the aggregation substance (Agg), *Enterococcus faecalis* endocarditis-associated antigen A (EfaA), and adhesion of collagen of *E. faecalis* (Ace) have been studied for their significant role in the biofilm formation.

Bacteriophages showed very promising results as alternative antibacterial agents for combating and controlling pathogenic bacteria **(**Moye et al. 2018). Noteworthy, only small doses of phages are needed to eradicate pathogenic bacteria, and this is due to the main characteristics of phages including propagation, self-replication, and high multiplication of phages **(**Bolocan et al. 2019). The effectiveness of bacteriophages to treat *E. faecalis* is due to their abilities to reduce tyramine and putrescine and their applications in cheese were assessed in previous studies **(**del Rio et al. 2019; 2021).

Bacteriocins are considered another antimicrobial approach that can be used to reduce food contamination by pathogenic bacteria to extend the food shelf life and they are ribosomally synthesized **(**Rendueles et al. 2022). The synergy between bacteriophage and bacteriocin can be achieved, and the combination of both has been used to control pathogenic bacteria such as *Listeria monocytogenes***(**Komora et al. 2020), *Staphylococcus aureus***(**Duc et al. 2020), *Salmonella***(**Yüksel et al. 2018), and *Clostridium perfringens***(**Heo et al. 2018). In this study, a novel *E. faecalis* phage, named vB_EfKS5, was isolated from cow feces and compost samples collected in Japan. Host range and whole-genome sequencing of the isolated phage were assessed. The ability of phage vB_EfKS5 to inhibit the growth of planktonic cells of *E. faecalis* and to destroy the biofilms was assessed. The synergy between phage vB_EfKS5 and nisin was evaluated in broth and milk and the results highlight the potential application of phage vB_EfKS5 or/ and nisin to inhibit the growth of *E. faecalis* to extend the shelf life of food and ensure food safety.

## Materials and methods

### Bacterial isolation and identification

*E. faecalis* was isolated from different food samples including cheese, vegetables, yogurt, and milk following the method described previously **(Chingwaru and Gashe **2003**).** Briefly, 25 g of samples were mixed with 225 ml of peptone water in a stomacher bag and homogenized for 2 min. After that, samples were serially diluted, and 0.1 ml of each sample was spread on the surface of bile esculin azide agar media **(BEA, Sigma-Aldrich, St Louis, MO).** For milk samples, 1 ml of milk was added to 9 ml of 0.1% peptone water **(Oxoid, UK)** in a sterile test tube and 0.1 ml of each dilution was inoculated onto BEA media. The inoculated plates were incubated at 37 °C for 24 h. The identity of the bacterial isolates and further confirmation by the polymerase chain reaction (PCR) was done. The PCR amplification of the 16 S rRNA gene was carried out using the following primers; Forward: AGAGTTTGATCCTGGCTCAG and reverse: GGTTACCTTGTTACGACTT. The PCR mixture included 5 µL Go Taq Green Master Mix×2 **(Promega, Madison, WI, USA)**, 1 µl of each primer, 1 µl of template DNA, and 3 µL distilled water. The TaKaRa PCR Thermal Cycler Dice **(Takara Bio Co., Tokyo, Japan)** was used to conduct the reactions. The condition of the PCR was as follows: denaturation at 95 °C for 5 min, 30 cycles of denaturation at 94 °C for 30 s, annealing at 51 °C for 30 s, and extension at 72 °C for 90 s, and a final extension at 72 °C for 5 min, and hold at 4 °C. The amplified products were electrophoresed on a 1.5% gel and stained with Midori Green Advance DNA stain **(Nippon Genetics Co., Ltd., Tokyo, Japan)**, and LuminoGraph 1 **(ATTO Co., Ltd., Tokyo, Japan)** was used to visualize the DNA. The PCR products were then purified using a PCR purification kit **(Nippon Genetics)** and sequenced for confirmation of isolates by comparing the sequence of the 16 S rRNA gene to those of *E. faecalis* or *E. faecium* in the database.

**Detection of*E. faecalis*virulence genes**.

A total of three genes encoding virulence factors including aggregation substance (*Asa1*), collagen-binding protein (ace), and *E. faecalis* endocarditis antigen (*efaA*) were detected using PCR as described before **(**Creti et al. 2004). The specific primers, amplicon sizes, and targeted genes are listed in **Table ****S1** The reaction of each gene was performed in a final volume of 25 µl using a TaKaRa PCR Thermal Cycler Dice. The amplified products were electrophoresed on a 1.5% gel and visualized as mentioned described above.

### Antibiotic susceptibility profile of *E. faecalis*

The antimicrobial susceptibility to 9 antibiotics was performed using the disk diffusion method according to the Clinical and Laboratory Standards Institute 2021 recommendations (CLSI). The antibiotic disks include penicillin (PEN), erythromycin (EM), gentamycin (GM), kanamycin (KM), rifampin (RM), vancomycin (VCM), ampicillin (ABP), amoxicillin-clavulanic acid (ACV), and ciprofloxacin (CIP). Briefly, 0.1 ml from overnight bacterial culture after dilution aseptically in TSB (optical density at 600 nm (OD_600_) value = 0.2) was swabbed using sterile cotton swabs on Müller-Hinton agar plates **(Nissui Pharmaceutical Co., Ltd., Tokyo, Japan)**, and antibiotic disks were aseptically added on the surface of the plates using sterile forceps, and the inoculated plates were incubated at 37 °C for 24 h. The results were interpreted as susceptible (S), intermediate resistance (IR), and resistant (R) according to CLSI guidelines (CLSI 2021**)**.

### Biofilm formation assay

The ability of *E. faecalis* isolates to form biofilms was estimated using the 96-microtiter plates method (Stepanoviće et al., 2007). Briefly, 200 µl of the bacterial culture (OD_600_ ~ 0.4) was inoculated in the wells of the plates and incubated for 24 h. After that, the wells were washed 3 times using sterile phosphate-buffered saline (PBS; 137 mM NaCl, 8.10 mM Na_2_HPO_4_, 2.68 mM KCl, 1.47 mM KH_2_PO_4_) and left to dry for 15 min. For fixation, 200 µl of 100% methanol was added to each well for 30 min. After removing methanol, the adherent biofilms were stained with 200 µl of 0.1% crystal violet for 30 min. Then, the plates were washed at least 3 times with sterile PBS and air-dried for 15 min. Then, 100 µl of 99.9% ethanol was added to solubilize the stain, and then the absorbance values at 595 nm (A_595_) were measured using an absorbance microplate reader (Infinite F50 Plus, Tecan, Japan) **(**Chajęcka-Wierzchowska et al. 2016). Blank wells contained tryptone soya broth **(TSB; Oxoid, Basingstoke, UK)** without any bacteria.

### Bacteriophages isolation and enrichment

Bacteriophages were isolated from 16 different samples including chicken feces, cow feces, compost, and raw milk collected from the Kyushu University farm, in Fukuoka, Japan. Briefly, 50 g of each sample was mixed with 100 µl of each *E. faecalis* isolate in a stomacher bag containing 100 ml of 2× TSB with 10 mM CaCl_2_ for 2 min and then incubated at 37 °C for 24 h. After that, 10 ml of the incubated suspensions were centrifuged at 12,000 ×*g* for 20 min at 4 °C, and the supernatant was filtrated using a 0.22 μm filter **(Merck Millipore, Ireland)** and used as a crude phage source. The detection of phages in the filtrated supernatant was done using the double-layer agar technique (Adams 1959). Briefly, 3ml of molten top agar (**Oxoid)** was inoculated with 100 µl of overnight bacterial culture and poured onto the surface of tryptone soya agar **(TSA; Oxoid).** Then, 15 µl of the phage suspension was spotted twice onto the multi-layer agar media, and plates were incubated at 37 ºC overnight. The following day, the plates were checked for the presence of lytic zones.

### Purification and propagation of bacteriophages

Isolated bacteriophages were purified and propagated from a single plaque. Briefly, a single pure plaque was picked up using a sterile micropipette tip and suspended in a microcentrifuge tube containing 1 ml of saline magnesium (SM) buffer (0.05 M Tris-HCl buffer, 0.1 M NaCl, 8 mM MgSO_4_, and 0.01% gelatin, pH 7.5). Then, 100 µl of serially diluted SM buffer containing the resuspended plaque was mixed with 100 µl of the host culture, added to 4 ml top agar, and then poured onto TSA plates. Then, the plates were incubated overnight at 37 ºC. The isolation of phage from a single plaque was repeated at least 3 successive times until homogenous plaque morphology was obtained to produce a purified phage stock. After purification, bacteriophages were propagated to obtain high-titer phage stocks using the plate lysate method **(**Bonilla et al. 2016).

### Host range and efficiency of plating (EOP) determination

The host range of our isolated phage was tested using the spot testing assay against 29 *E. faecalis* and 7 *E. faecium* hosts. Briefly, the top agar inoculated with 100 µl of the bacterial host was poured onto TSA solid plates and left to dry. Then, 10 µl of phage was spotted on the bacterial lawn and incubated at 37 ℃ for 24 h to check the lytic activity. The effectiveness of phage vB_EfKS5 against all sensitive *E. faecalis* host isolates was further assessed by the efficiency-of-plating method (EOP using the spot test assay as described before (Khan Mirzaei and Nilsson 2015**).** Briefly, the phage was serially diluted 10-fold in SM buffer. The top agar was inoculated with 100 µl of the fresh culture of each bacterium and poured onto the TSA plates, and 10 µl of each dilution was spotted in triplicates. The plates were incubated at 37℃ for 24 h to calculate the phage titer. EOP has been calculated as the ratio of the average PFU on target bacteria/average PFU on host bacteria.

### Temperature and pH stability assays

Phage vB_EfKS5 stability was assessed at different temperatures (40, 50, 60, 70, 80, 90, and 100ºC) over 1 h using the method described by Hammerl et al. 2014). Phage vB_EfKS5 was incubated for 1 h in a water bath set at each temperature and 100 µl of the phage suspension was withdrawn at 10 min intervals, diluted, and immediately plated for phage titration. The stability of phage vB_EfKS5 at different pH values ​​was monitored **(**Park et al. 2017). The pH values of 2, 3, 4, 5, 6, 7, 8, 9, 10, 11, 12, and 13 were prepared in PBS buffer and adjusted with 1 M HCl or 1 M NaOH solutions. Phage was incubated overnight at room temperature in each pH tube and the stability of the residual phage was assessed by plaque assay. Both temperature and pH experiments were conducted in triplicates.

### Bacteriolytic activity and one-step growth analysis

The optimum Multiplicity Of Infection (MOI) of phage vB_EfKS5 was determined using the method described before **(**Li et al. 2021). The MOI is defined as the ratio of phage (PFU) titer to the number of the host bacteria (CFU). Briefly, 1 ml of phage vB_EfKS5 was mixed with an equal volume of the *E. faecalis* host at different MOIs (0.01–100), and the mixture was incubated at 37 °C for 3 h with shaking (160 rpm). After that, samples were withdrawn and centrifuged at 10,000 × *g* for 10 min. and the supernatant was serially diluted in SM buffer and spotted onto a double-layer agar plate aseptically to determine the phage titer. The MOI which gave the highest reduction in bacterial titer was considered the optimal MOI. The one-step growth curve of phage vB_EfKS5 was determined using the method described by Kropinski (2018**).** Briefly, *E. faecalis* isolate no.7 was grown to OD_600_ ~ 0.2 and infected with vB_EfKS5 phage at MOI of 0.1, and then the mixture was allowed to adsorb for 7 min at 37 °C. After adsorption, the mixture was centrifuged at 10,000 × *g* for 10 min. The supernatant was removed, and the pellet was resuspended in 10 ml sterile TSB and incubated at 37 °C for 90 min. Then, 200 µl was taken every 10 min, centrifuged, serially diluted in SM buffer, and immediately plated for phage titration. The experiment was repeated 3 times to calculate the latent period and burst size.

### Bacteriolytic activity of phage against planktonic *E. faecalis* and its antibiofilm activity

The inactivation of *E. faecalis* planktonic cells and the antibiofilm activity with vB_EfKS5 phage was assessed in TSB broth in 96-well microtiter plates at different MOIs **(**Lerdsittikul et al. 2022). For the planktonic cells, the *E. faecalis* culture (1 × 10^8^ CFU/ml) was mixed with phage suspension at different MOIs (0.001–1000) and the plates were incubated at 37 °C for 24 h and the growth of bacteria was estimated every 4 h by measuring absorbance at 595 nm.

The antibiofilm efficacy of phage vB_EfKS5 against *E. faecalis* was assessed using a 96-microtiter plate method as previously described **(**Sharma et al. 2021). Briefly, the plate wells were inoculated with the bacterial culture after dilution in TSB (OD_600_ ~ 0.2) and incubated at 37 °C for 24 h. The next day, the unattached cells were removed, and the wells were washed 3 times with PBS and dried in air. Subsequently, the phage vB_EfKS5 was added to the wells containing bacterial biofilm at different MOIs (0.01, 0.1, and 1) and incubated overnight at 37 °C. The supernatant was removed, and the wells were gently washed 3 times with PBS. Then, the plates were stained with 0.1% crystal violet solution for 20 min. After the removal of stains, wells were washed again with PBS. The ethanol (99.9%) was added to each well, and the absorbance was measured at A_595_ using an absorbance microplate reader.

### Whole genome sequencing and bioinformatic analysis

The phage vB_EfKS5 genomic DNA was extracted from the purified high titer phage suspension (10^11^ PFU/ml) using the High Pure Viral Nucleic Acid Kit (Roche, Mannheim, Germany). The library preparation and whole-genome sequencing were carried out by Novogene (Japan). The whole genome was sequenced using an Illumina HiSeq system. The read sequences were assembled using DFAST v. 1.2.18 **(**Tanizawa et al. 2018). The assembled genome was annotated using the RAST server **(**Aziz et al. 2008), and further confirmation was done by BLAST analysis (Altschul et al. 1997). tRNA genes were identified using tRNAScan-SE v2.0 **(**Lowe and Eddy 1997**).** BLASTN and BLASTP programs were run to assign possible functions to the ORFs (Altschul et al. 1997). A phylogenetic tree of phage VB_EfKS5 with other related *Enterococcus* phages was created using Geneious v8.1.2 (https://www.geneious.com).

### Determination of MIC and MBC of Nisin against *E. faecalis*

A fresh stock solution of nisin **(10**^**6**^**IU/g; Sigma, MO, USA)** in 0.02 N HCl containing 0.75% NaCl was prepared and sterilized using a sterile 0.22-µm pore size membrane filter **(Merck Millipore)** before the experiment and then diluted two-fold (4000, 2000, 1000, 500, 250, 125, 62.5 U/ml) with sterile PBS. The overnight culture of *E. faecalis* JCM 7783 was adjusted to ~ 10^6^ CFU/ml in TSB and the wells were inoculated with 100 µl of nisin and 80 µl of TSB. The bacterial suspension (20 µl) was added to each well and the control contained only 100 µl of water instead of nisin and 80 µl of TSB. The plates were incubated for 18–24 h at 37 °C. To determine the MBC, 10 µl of the corresponding inhibitory concentration was spotted aseptically on the agar plate in duplicates. After overnight incubation, the MBC was observed and defined as the lowest concentration that inhibited the visible growth of the subculture.

### The combined effect of phage and nisin in broth

The efficiency of phage alone or in combination with nisin against planktonic *E. faecalis* in broth was assessed by the method previously described **(**Duc et al. 2020). Briefly, four groups were designed as follows:

Group A (Control): TSB (4700 µl) + *E. faecalis* (10^5^ CFU, 100 µl) + PBS (200 µl).

Group B: TSB (4700 µl) + *E. faecalis* (100 µl) + phage (10^5^ PFU, 100 µl) + PBS (100 µl).

Group C: TSB (4700 µl) + *E. faecalis* (100 µl) + nisin (500 U/ml, 100 µl) + PBS (100 µl).

Group D: TSB (4700 µl) + *E. faecalis* (100 µl) + phage (100 µl) + nisin (100 µl).

All mixtures were incubated at 37 °C for 24 h and 100 µl of each group was collected every 2 h, serially diluted, and spotted on TSA plates to enumerate the total viable count.

### Synergistic antibacterial effect of phage and nisin in milk

The ability of vB_EfKS5 phage and nisin as well as the combination of both to inhibit the growth of *E. faecalis* in pasteurized milk was also estimated. Approximately 10^5^ CFU/ml of *E. faecalis* was inoculated in 5 ml of milk and the phage was added at MOI of 1 with/without nisin (500 U/ml), and then the mixture was incubated at 37 °C for 24 h. The control group was treated only with sterile PBS without phage or nisin. The viable cells of the *E. faecalis* isolates were counted every 2 h as described above and are presented as CFU/ml.

### Statistical analysis

All experiments were conducted three times. Data were analyzed statistically using GraphPad prism version 8.0.0 and were expressed as the mean ± standard deviation (SD) or percentage (%). The significance among the different groups was estimated using Student’s *t-test*. A *p*-value lower than 0.05 was considered significant.

## Results

### Isolation of *E. faecalis* and the presence of the virulence genes

A total of 29 *Enterococcus* spp. (19.33%) isolates were isolated from 150 food samples. Of the 29 isolates, 28 (96.6%) were confirmed as *E. faecalis*, and one isolate (3.4%) was confirmed as *E. faecium*. A total of three virulence genes were detected among *E. faecalis* isolates and the results showed that 89.28% of isolates have *efaA*, 78.57% of isolates have *ace* and 21.42% of *E. faecalis* isolates encode the *asa1* gene (Fig. S1). DNA prepared from *E. faecalis* JCM 7783 was used as a positive control template. **(Table S2).**

### Antibiotic susceptibility of *E. faecalis*

**Table S3** shows the sensitivity of *E. faecalis* isolates to 9 commonly used antibiotics. The majority of *E. faecalis* isolates exhibited resistance to kanamycin (75%) and rifampin (53.57%). Of the 28 *E. faecalis* isolates, 5 (17.85%) were susceptible to gentamycin, 18 isolates (64.28%) were intermediate-resistant, and 5 isolates were resistant (17.85%). The resistance rates to ciprofloxacin and erythromycin were 25% and 14.28% respectively, while 39.28% and 67.85% showed intermediate resistance to both antibiotics, respectively. All isolates were resistant to penicillin and vancomycin; except 10.71% and 14.28% of isolates showed intermediate resistance to both antibiotics, respectively. No resistance was detected to ampicillin and amoxicillin-clavulanic acid (Fig. S3).

### Biofilm formation ability of *E. faecalis* isolates

The absorbance results at 595 nm show that most *E. faecalis* isolates could form biofilm. Half (50%) of the *E. faecalis* isolates showed a strong ability to form biofilms, 42.3% formed moderate biofilms, and only 7.69% exhibited weak biofilms (**Fig. S3**).

### Isolation and host range of phage vB_EfKS5

Five bacteriophages (vB_EfKS1, vB_EfKS2, vB_EfKS3, vB_EfKS4, and vB_EfKS5) out of the 16 different samples were isolated using the double-layer agar technique. Ten *E. faecalis* isolates (*E. faecalis* isolates numbers: 2,3,5,7,9,10,16,27,22,23) were used as a host for isolating and propagating the possible bacteriophage candidates. Phage vB_EfKS5 was selected for further experiments based on its capacity to infect a wide range of *E. faecalis* isolates by exhibiting repeatable lytic zones and yielding high titers when propagated on the targeted hosts **(Fig. S4).** This phage was plaque purified many times and propagated to produce high titer stocks by using the plate lysis methodology. The host ranges of phage vB_EfKS5 were determined using a collection of 29 *E. faecalis* isolates and 7 *E. faecium* isolates. As indicated in Table 1, phage vB_EfKS5 had a broad host range since it infected 75.86% (22/29) of *E. faecalis* hosts and 3 out of 7 (42.85%) *E. faecium*. These results highlight the high effectiveness and broad spectrum of phage vB_EfKS5 against *Enterococcus* spp.

**Table 1:** Host range of phage vB_EfKS5 against *Enterococcus* sp

*Enterococcus sp.*	Phage vB_EfKS5
*E. faecalis* 1	
*E. faecalis* 2	+
*E. faecalis* 3	+
*E. faecalis* 4	+
*E. faecalis* 5	+
*E. faecalis* 6	+
*E. faecalis* 7	+
*E. faecalis* 8	+
*E. faecalis* 9	+
*E. faecalis* 10	
*E. faecalis* 12	+
*E. faecalis* 13	+
*E. faecalis* 14	
*E. faecalis* 15	
*E. faecalis* 16	+
*E. faecalis* 17	
*E. faecalis* 18	+
*E. faecalis* 19	+
*E. faecalis* 20	+
*E. faecalis* 21	
*E. faecalis* 22	+
*E. faecalis* 23	+
*E. faecalis* 24	
*E. faecalis* 25	+
*E. faecalis* 26	+
*E. faecalis* 27	+
*E. faecalis* 28	+
*E. faecalis* 29	+
*E. faecalis* JCM 5803	+
*E. faecium* 20	
*E. faecium* 22	+
*E. faecium* 23	
*E. faecium* 24	
*E. faecium* 25	
*E. faecium* 30	+
*E. faecium* JCM 5804	+

-: negative; +: positive

### Efficiency of plating of vB_EfKS5 phage

All the *E. faecalis* hosts that are infected by vB_EfKS5 phage were used to estimate the phage productivity. The *E. faecalis* isolate no. 7 was the original host, and the other susceptible *E. faecalis* hosts were considered indicator isolates. The results showed that 70% of the isolates were ≥ 0.5 which reveals the high production of isolated phage, while 30% of isolates showed medium production of phage vB_EfKS5 **(Table S4).**

### Temperature and pH stability of vB_EfKS5 phage

**Fig. 1:**
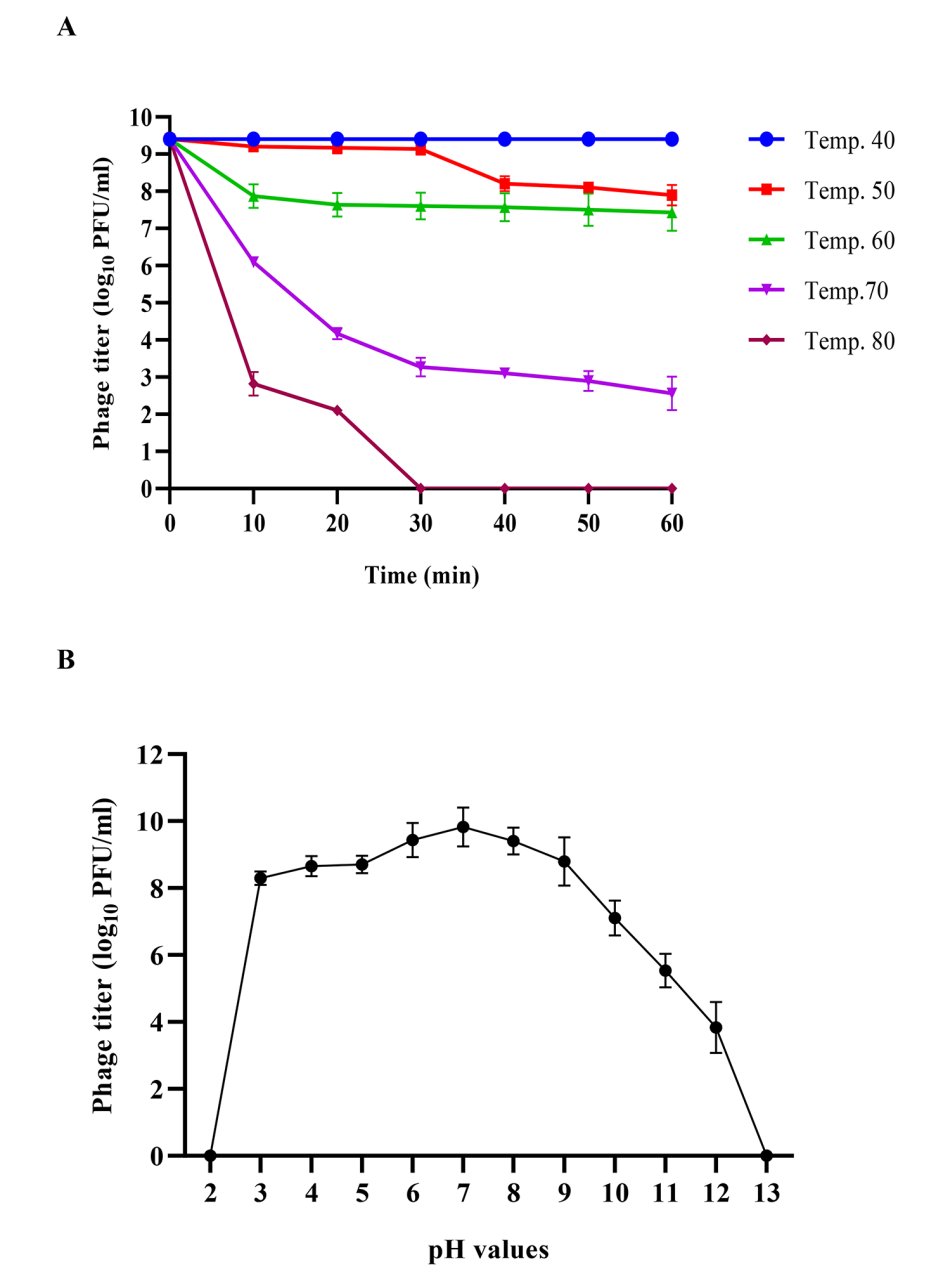
Effects of temperature and pH on the stability of phage vB_EfJKS5. (**A**) Phage vB_EfKS5 was exposed to different temperatures 40 to 100 °C for 1 h, and the phage titers were measured at 10 min intervals. (**B**) phage was incubated at different pH values for 24 h. Phage titers are recorded as means ± standard deviation

For any phage biocontrol applications, it is very important to evaluate the phage stability under different harsh conditions. The stability of phage vB_EfKS5 at different temperatures ranging from 40 to 100 °C was studied. The isolated phage showed high activity and infectivity at various temperatures. The phage titers of 10^9^ PFU/ml were stable at a temperature of 40 ºC for 1 h. At 50 ºC, the phage titer decreased from 10^9^ to 10^8^ PFU/ml after 30 min and remained stable at 10^8^ PFU/ml at 60 ºC for 1 h. The phage titer was reduced to 10^3^ PFU/ml at 70 ºC and to 10^2^ PFU/ml after 20 min at 80 ºC. After 30 min at 80 ºC, the phage could not tolerate the temperature and was not recovered **(**Fig. 1a**).** The stability of phage vB_EfKS5 at different pH values is shown in Fig. 1b. The highest phage titer was observed at pH 7 and the titer was approximately 10^9^ PFU/ml. The phage was still active at pH 12 while its activity was reduced, not active, at pH 2 and pH 13.

### Optimum MOI and one-step growth curve of vB_EfKS5 phage

**Fig. 2:**
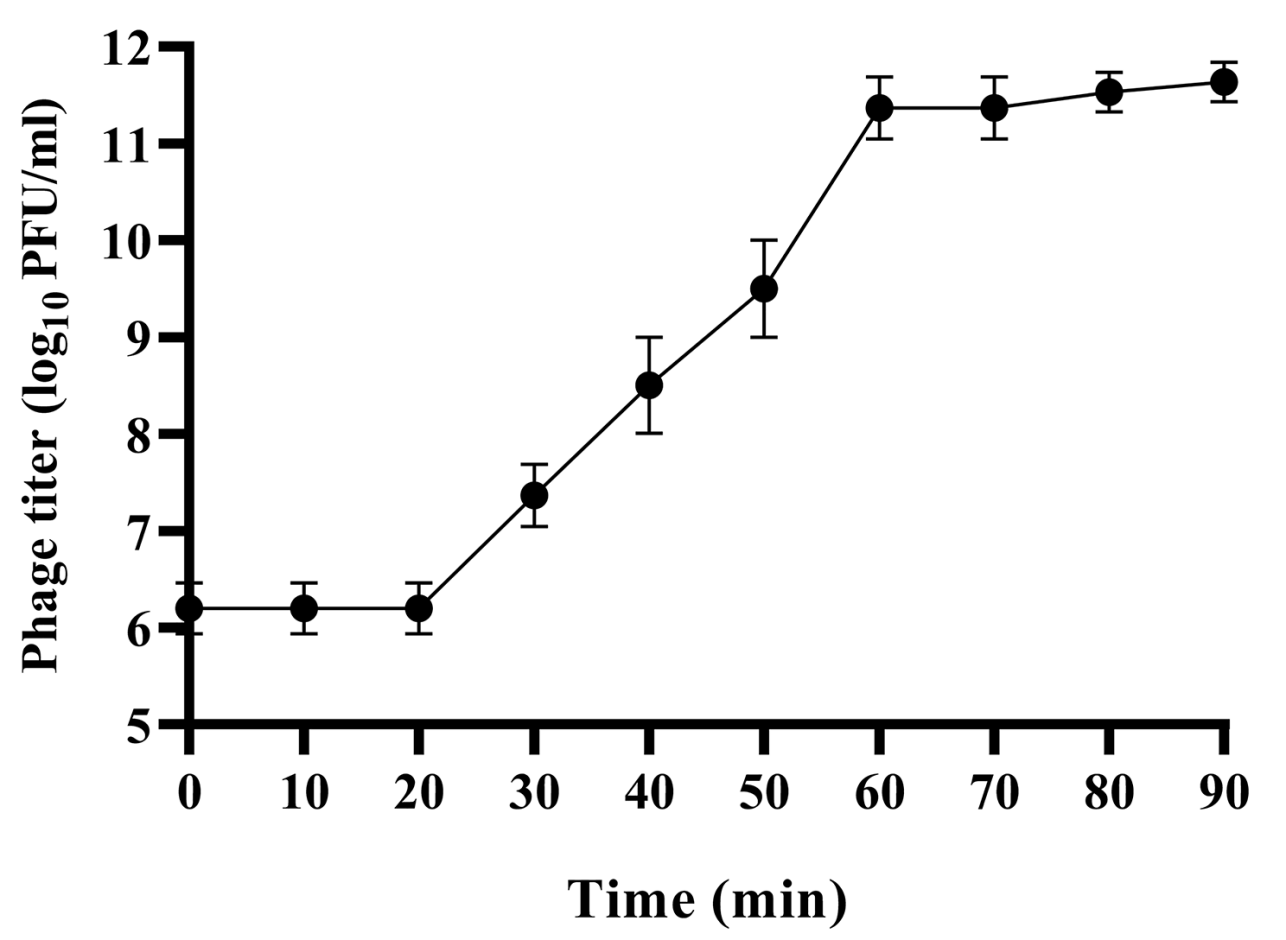
Growth curve of phage vB_EfKS5 showing the latent period and burst size. Experiments were repeated three times with duplicate samples

For any phage application, it is important to find out the optimum MOI. Accordingly, different titers of phage were mixed with *E. faecalis* host to achieve different MOIs. At MOI of 1, the vB_EfKS5 phage titer reached the highest value compared to other MOIs, indicating that MOI of 1 was the optimum MOI **(data not shown).** The one-step growth curve showed that the latent period was estimated to be 20 min and the phage titers increased significantly over the next 50 min, followed by steady growth until 90 min. The average burst size of the vB_EfKS5 phage was calculated to be approximately 183.33 PFU per infected cell **(**Fig. 2**).**

**Bacterial lysis and antibiofilm efficiency of vB_EfKS5 phage against*E. faecalis***.

**Fig. 3:**
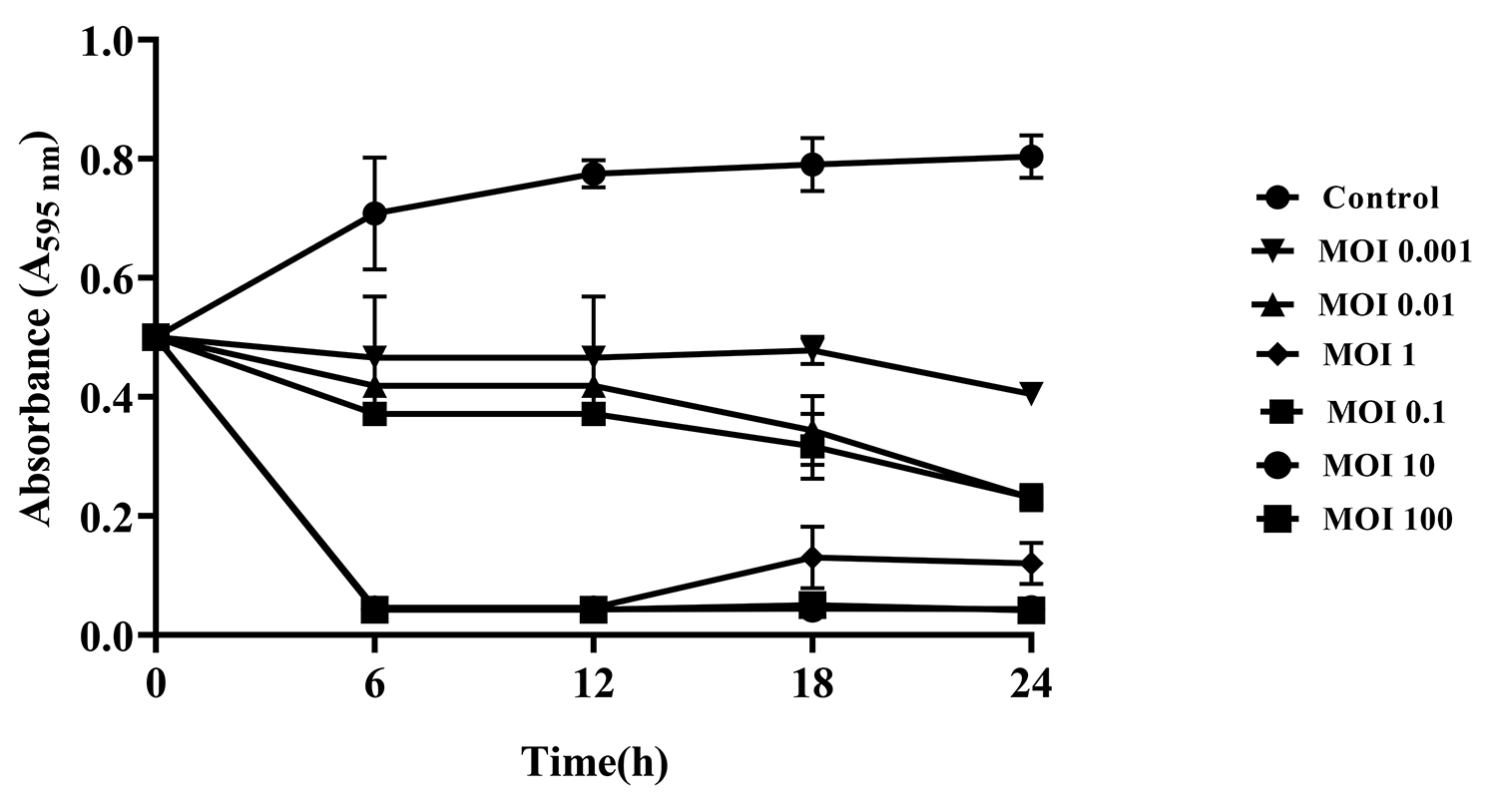
Bacterial killing activity of phage vB_EfKS5 against host cells at different MOI (0.001- 100) in TSB medium. Experiments were run in three replicates; the presented data are the average of these replicates ± standard deviation

**Fig. 4:**
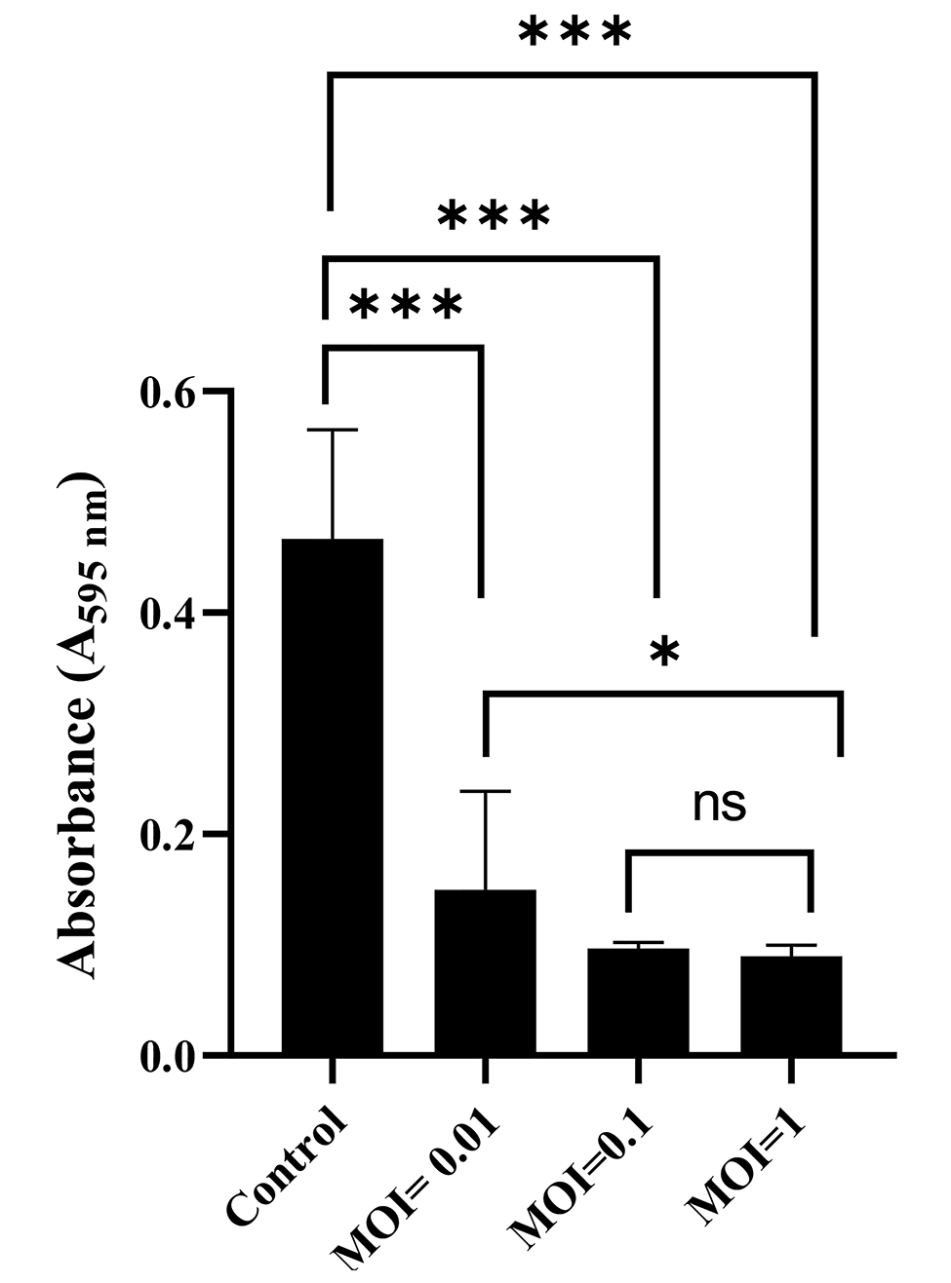
Anti-biofilm activity of phage vB_EfKS5 at different multiplicities of infection (MOI). Stars indicate a significant difference between variables where: (*P < 0.05) and (***P < 0.001), and ns indicates non-significance. Experiments were run in three replicates; the presented data are the average of these replicates ± standard deviation

The antibiofilm activity of phage vB_EfKS5 was investigated at different MOIs in TSB and the OD_600_ values ​​were measured every 4 h for 24 h. Results showed a significant decrease in optical density over time compared to the controls that showed steadily increased growth during the 24 h incubation. At the highest MOIs such as 10, 100, and 1000, no bacterial growth was detected. Bacterial growth was also significantly (*P ˂ 0.05*) reduced when treated with low MOIs (0.001, 0.01, and 0.1) **(**Fig. 3**).** These results revealed that vB_EfKS5 phage could effectively inhibit the growth *E. faecalis* of *E. faecalis* with different MOI ranges (0.001–1000). The biofilm of *E. faecalis* No.7 was established and then challenged with vB_EfKS5 phage at different MOIs. The results (Fig. 4**)** showed that the content of crystal-stainable biofilms after treatment with phage was significantly reduced at all tested MOIs compared to controls. The MOI of 1 had the greatest effect, as it significantly reduced (*P ˂ 0.05*) A595 readings compared to controls, followed by MOIs of 0.1 and 0.01.

### Genomic analysis of vB_EfKS5 phage

**Fig. 5:**
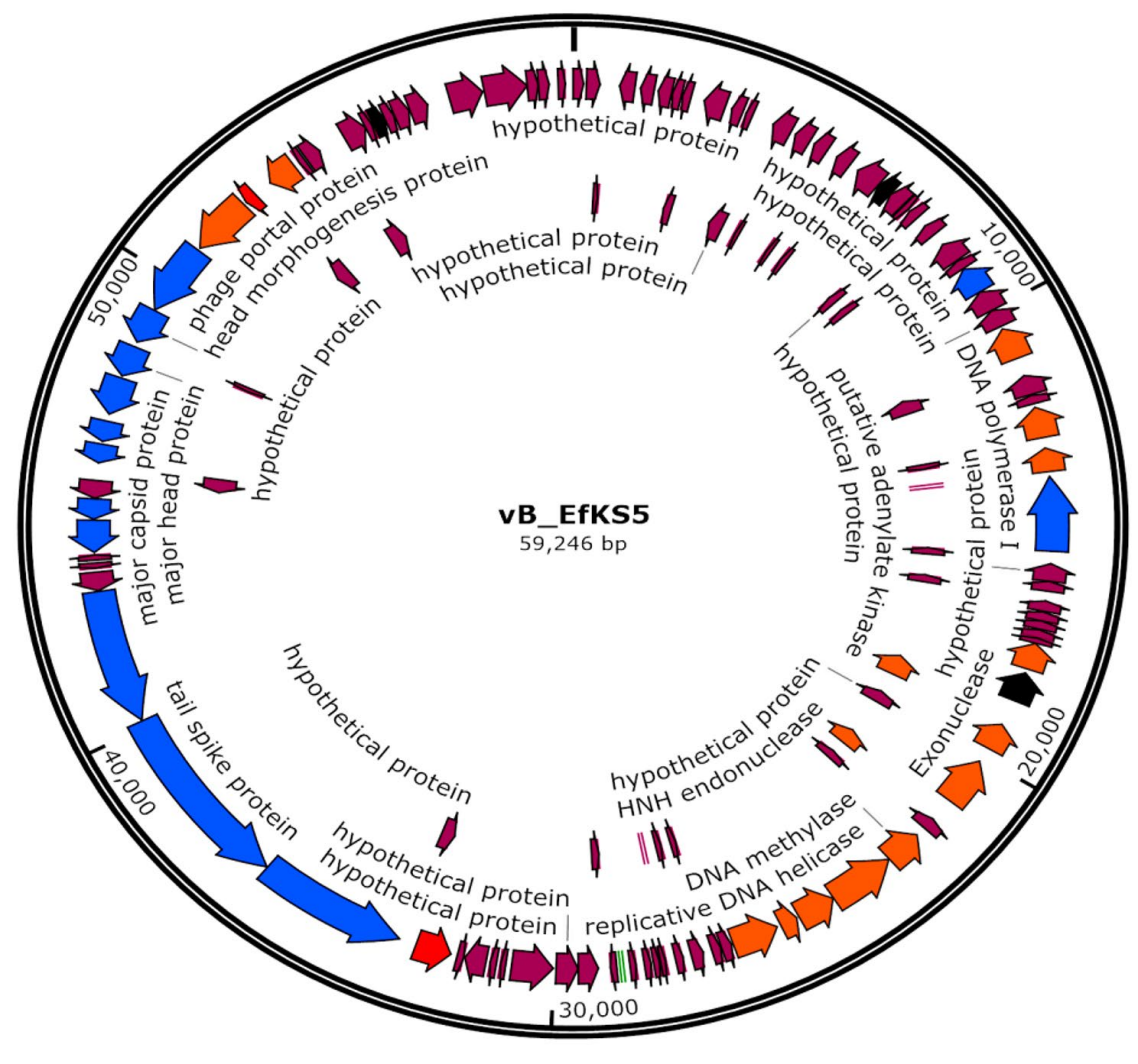
Genome map of phage vB_EfJKS5. The genome size of vB_EfKS5 was identified as 59,246 bp, and 125 putative ORFs were predicted. Violet arrows represent predicted hypothetical proteins, blue represents structural proteins, orange represents nucleotide regulation, red represents host lysis proteins, and black represents other functions

The genome of phage vB_EfKS5 (GenBank Accession Number: OQ297175), a circular genome, is 59,246 bp in length, has 125 predicted ORFs, one tRNA gene, and a G + C content of 39.75%. The nucleotides composition in the phage genome is as follows: G (10,894 bp, 18.39%), C (12,656 bp, 21.36%), A (16,180 bp, 27.31%), and T (19,516 bp, 32.94%). 91 ORFs (72.8%) were designated as hypothetical proteins, while only 34 ORFs (27.2%) were predicted to be functional proteins **(**Table 2**).** Using RAST and BLASTP, four functional groups were identified: morphogenesis-related proteins, DNA replication and manipulation, lysis of host cells, and other proteins related to other functions **(**Fig. 5**).**

**Table 2:** List of functionally annotated proteins from ORFs in the genome of phage vB_EfKS5

ORF	Predicted function	Position (5’-3’)	Amino acid sequence identity/similarity to best homologs	GenBank Accession no.
ORF 22	Phage DNA binding protein	6512–6817	Enterococcus phage IMEEF1: Phage DNA binding protein	YP_009603915.1
ORF 31	Tail-length tape-measure protein	8968–9552	Enterococcus phage vB_EfaH_EF1TV: putative cytidine deaminase/tail length tape-measure protein	AZV00019.1
ORF 34	ATP-dependent metalloprotease	10,454 − 11,149	Enterococcus phage SAP6: ATP-dependent metalloprotease	YP_009604001.1
ORF 38	DNA polymerase I	12,246 − 12,914	Enterococcus phage IMEEF1: DNA polymerase	YP_009603915.1
ORF 40	HNH homing endonuclease	13,136 − 13,648	Enterococcus phage EFap05-1: HNH homing endonuclease	UIE13835.1
ORF 42	DNA polymerase I	13,747 − 15,345	Streptococcus phage SP-QS1]:DNA polymerase I	YP_008320536.1
ORF 52	LPS glycosyltransferase	17,341 − 17,904	Enterococcus phage EfsWh-1: LPS glycosyltransferase	QAY01541.1
ORF 53	Putative sigma factor	17,966 − 18,628	Enterococcus phage vB_EfaS_TV16: putative sigma factor	QIG60321.1
ORF 54	Putative adenylate kinase	18,621 − 19,190	Enterococcus phage phiM1EF2: adenylate kinase	BCF74614.1
ORF 55	Crossover junction endodeoxyribonuclease RuvC	19,187 − 19,765	Enterococcus phage phiM1EF: putative crossover junction endodeoxyribonuclease RuvC	BCF74615.1
ORF 57	Exonuclease	20,087 − 21,115	Enterococcus phage: Entf1: exonuclease	QDB70560.1
ORF 58	HNH endonuclease	21,108 − 21,548	Enterococcus phage phiM1EF2: HNH homing endonuclease	BCF74618.1
ORF 61	DNA methylase	22,083 − 22,835	Enterococcus phage EFap05-1: DNA methylase	UIE13815.1
ORF 62	DNA helicase	22,848 − 24,212	Streptococcus phage SP-QS1: replicative DNA helicase	YP_008320518.1
ORF 63	DNA replication protein	22,224 − 25,000	Enterococcus phage Entf1: DNA replication protein	QDB70565.1
ORF 64	Transcriptional regulator	25,094 − 25,402	Enterococcus phage IMEEF1: transcriptional regulator	YP_009603955.1
ORF 65	DNA primase	25,477 − 26,421	Enterococcus phage SAP6: DNA primase	YP_009604014.1
ORF 87	N-acetylmuramoyl-L-alanine amidase	32,053 − 32,766	Enterococcus phage vB_EfaS_HEf13: N-acetylmuramoyl-L-alanine amidase	AYH92708.1
ORF 89	Tail spike protein	33,114 − 36,140	Enterococcus phage EF653P1: tail spike protein	WAX15251.1
ORF 90	Tail fiber protein	36,153 − 40,145	Enterococcus phage vB_EfaS_HEf13: tail fiber protein	AYH92711.1
ORF 91	Tail-length tape-measure protein	40,159 − 43,044	Enterococcus phage vB_EfaS_HEf13: tail length tape-measure protein	AYH92712.1
ORF 95	Major tail protein	43,876 − 44,565	Enterococcus phage BC611: major tail protein	YP_006488747.1
ORF 96	Tail terminator	44,586 − 45,020	Streptococcus phage SP-QS1: tail terminator	YP_008320490.1
ORF 99	Head-tail connector family protein	45,791 − 46,195	Enterococcus phage Entf1: head-tail connector family protein	QDB70499.1
ORF 100	Major tail protein	46,255 − 46,695	Enterococcus phage vB_EfaS_HEf13: major tail protein	AYH92720.1
ORF 101	Major capsid protein	46,850 − 47,656	Enterococcus phage vB_EfaS_TV16: major capsid protein	QIG60313.1
ORF 102	Head scaffolding protein	47,705 − 48,373	Enterococcus phage vB_EfaS_IME198: head scaffolding protein	YP_009218884.1
ORF 104	Head morphogenesis protein	48,484 − 49,239	Enterococcus phage vB_EfaS_TV16: head morphogenesis protein	QIG60312.1
ORF 105	Phage portal protein	49,251 − 50,786	Enterococcus phage vB_EfaS_HEf13: Phage portal protein	AYH92724.1
ORF 106	Phage terminase large subunit	50,843 − 52,114	Enterococcus phage vB_EfaS_Ef7.1: Phage terminase large subunit	QBZ69408.1
ORF 107	Holin	52,177 − 52,425	Enterococcus phage EF-P29: holin	APU00267.1
ORF 109	Phage terminase small subunit	52,802 − 53,401	Enterococcus phage vB_OCPT_CCS2: terminase small subunit	UQT01010.1
ORF 114	Methyltransferase	54,554 − 55,021	Enterococcus phage SAP6: methyltransferase	YP_009603990.1
ORF 116	putative glutaredoxin	55,187 − 55,447	Enterococcus phage EfsWh-1: putative glutaredoxin	QAY01492.1

The phage vB_EfKS5 encodes a set of enzymes necessary for DNA replication, metabolism, and manipulation. For example, OFR 38 and ORF 42 encode DNA polymerase I, ORF 61 encodes DNA methylase, ORF 62 encodes DNA helicase, ORF 65 encodes DNA primase, ORF40 and ORF 58 encodes HNH homing endonuclease, ORF 52 encodes LPS glycosyltransferase, ORF 54 encodes adenylate kinase, ORF 57 encodes exonuclease, ORF 34 encodes ATP-dependent metalloprotease, ORF 114 encodes methyltransferase, and ORF 116 encodes putative glutaredoxin.

The phage structural module contains genes involved in the host recognition and phage structural assembly: tail-associated proteins (ORF 89, ORF 90, ORF 91, ORF 95, ORF 96, ORF 99, and ORF 100 encode tail spike protein, tail fiber protein, tail length tape-measure protein, head-tail connector, and major tail proteins), and head-associated proteins (ORF 101, ORF 102, and ORF 104 encode major capsid protein and head morphogenesis protein). DNA packaging proteins include the phage portal protein (105), the phage terminase large subunit (ORF 106), and the small subunit (ORF 109). There are two proteins involved in the host cell lysis modules: holin (ORF 107), and N-acetyl muramyl-L-alanine amidase (ORF 87).

In addition, the phage vB_EfKS5 does not encode any genes related to lysogeny, drug resistance, or toxin which suggests the safety of using this phage in food application and therapy. The homology of the vB_EfKS5 phage with other previous phages was investigated by running BLASTN **(**Table 3**).** Phylogenetic analysis was then created based on the whole genome sequences of vB_EfKS5 and other *Enterococcus* phages **(Fig. S5).** Phage vB_EfKS5 was observed to cluster specifically with the *Enterococcus* phage vB_EfaS_HEf13, which has a wide host range and therapeutic efficacy against clinical *Enterococcus* isolates **(**Lee et al. 2019).

**Table 3:** Homology of vB_EfKS5 phage with other related Enterococcus phages

Genome characteristics	Phages (GenBank Accession number)						
	vB_EfKS5 (OQ297175)	vB_EfaS_TV16 (MN939408.1)	vB_OCPT_PG2 (ON113177.1)	EF653P5 (OP172800.1)	vB_EfaS_HEf13 (MH618488.1)	VD13 (NC_041861.1)	IMEEF1 (NC_041959.1)
Genomic size (bp)	59,246	58,127	57,485	56,467	57,811	55,073	55,073
**GC content (%)**	**39.7**	**40.1**	**40.1**	**40**	**40**	**40**	**40**
**Per identity** **(%) with vB_EfKS5**	**100**	**95.39**	**95.17**	**94.32**	**96.3**	**93.2**	**94.42**
**Query coverage (%) with vB_EfKS5**	**100**	**90**	**89**	**88**	**90**	**79**	**89**

### Inactivation of *E. faecalis* growth in broth and milk

**Fig. 6:**
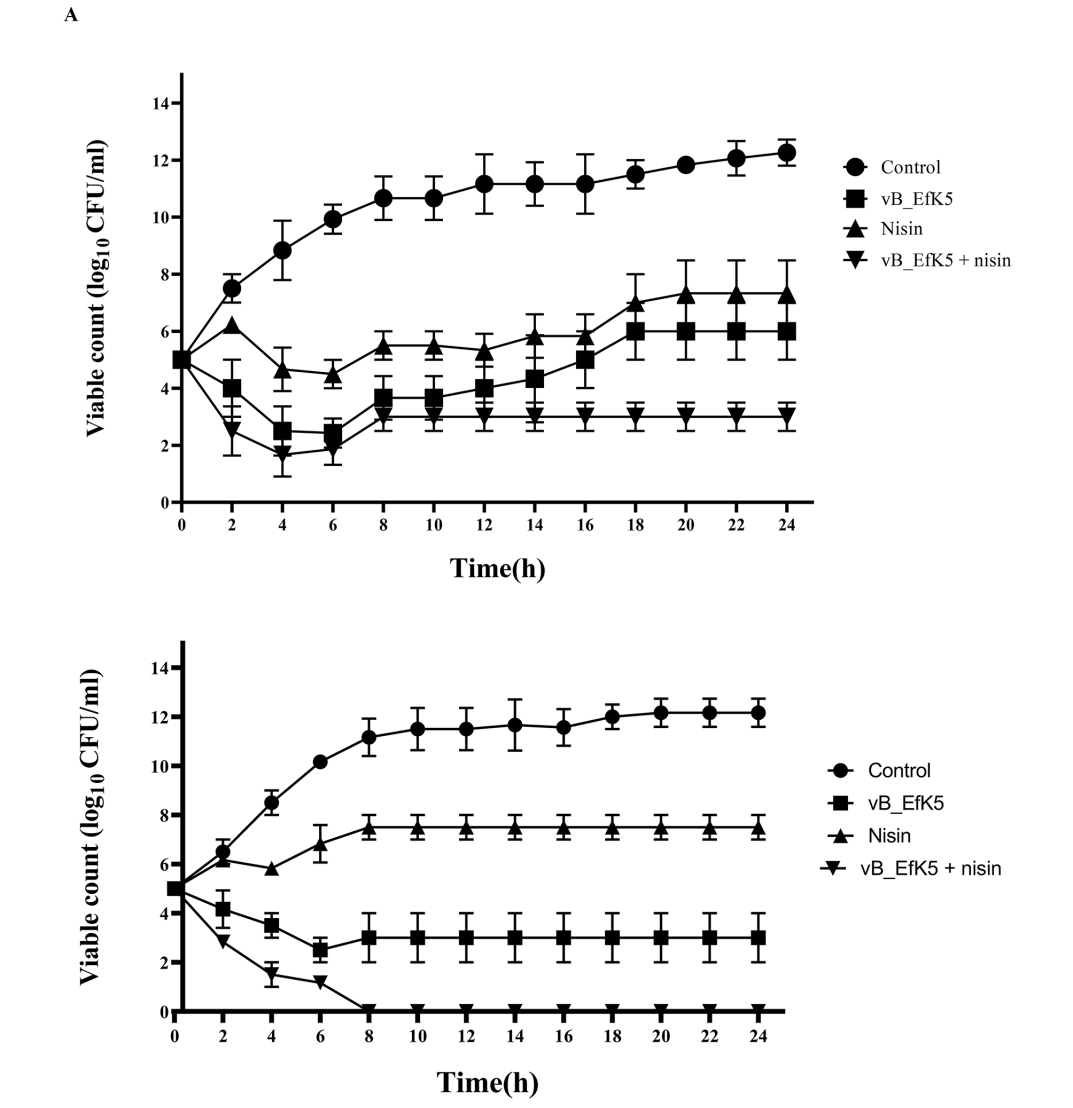
Effects of phage vB_EfKS5 alone or in combination with nisin on the viability of *E. faecalis* (A) in broth and (B) in milk. Error bars show the standard deviation of the mean (n = 3)

It was important to study the biocontrol activity of phage vB_EfKS5 against *E. faecalis* in food to evaluate its effectiveness in extending the shelf life and enhancing food safety. The MIC of nisin against *E. faecalis* isolates was 500 U/ml (0.5 mg/ml), and MBC was 1000 U/ml (1 mg/ml). Figure 6a shows the antibacterial effect of phage or/ and nisin against *E. faecalis* in TSB. The viability of *E. faecalis* treated with phage alone was significantly (*P ˂ 0.05*) reduced by 5 log_10_ CFU/ml compared to controls after 2 h of treatment. Nisin treatment demonstrated antibacterial activity after 4 h, where the viable counts decreased by 4 log_10_ CFU/ml compared to the control. A synergistic effect of phage and nisin was observed when nisin and phage were used in combination. Compared to controls, this combination reduced the bacterial counts by 8 log_10_ CFU/ml after 10 h of incubation. After 24 h, the treatment with nisin at 500 U/ml, the viable counts of *E. faecalis* were decreased (P < 0.05) by 4 logs compared with the control. The treatment with phage vB_EfKS5 alone caused significant reductions (P < 0.05) of *E. faecalis* counts by 6 logs at 24 h compared with the control. The combined use of phage vB_EfKS5 and nisin decreased (P < 0.05) the viable counts of *E. faecalis* by 9 logs after 24 h. The combination of phage and nisin was still more effective than individual antimicrobials since it delayed bacterial regrowth and the emergence of resistant bacteria. These results indicated a strong synergistic effect between phage and nisin, although phage alone has a strong antibacterial effect. The effect of phage alone or in combination with nisin on *E. faecalis* viability in whole pasteurized milk is shown in Fig. 6b. Nisin reduced viability (P ˂ 0.05) after 4 h by 2.5 logs, but the bacteria grew again afterward. The treatment with phage alone resulted in a 5-log reduction in bacterial counts compared to control (P ˂ 0.05). The combined use of phage and nisin extremely decreased viable counts (P ˂ 0.05) after 4 h by 6 logs compared to the control. After 24 h, the viable counts of *E. faecalis* in the treatment with phage and nisin in combination were reduced to below the detection limit.

## Discussion

*E. faecalis* can cause a variety of nosocomial (hospital-acquired infections) infections and is also considered a food contaminant that affects the taste and flavor of food **(**Franz et al. 2011; Hammerum 2012**).** Toxic compounds such as biogenic amines (tyramine and putrescine) can be produced by *E. faecalis*, and their accumulation can cause severe diseases due to their toxicity **(**Linares et al. 2011). Previous studies have proven the possible infection of *E. faecalis* to the human gastrointestinal tract through the consumption of contaminated food or raw milk with the bacteria **(Anderson et al. **2016**).** Furthermore, *E. faecalis* can integrate into oral biofilm after consumption of contaminated food such as cheese and this may lead to oral treatment failure **(**Al-Ahmad et al. 2010). Previously, we targeted multidrug-resistant *E. faecalis* isolated from an oral endodontic infection with phage vB_ΦZEFP, and the results demonstrated the potential application of this phage to prevent root canal treatment failure **(**El-Telbany et al. 2021). In this study, we characterized a novel phage named vB_EfKS5 and investigated its effect, alone or in combination with nisin, to control and inhibit the growth of *E. faecalis* isolated from various food types and investigated its possible application in food to control *E. faecalis* growth.

*E. faecalis* isolates were isolated from various food samples and some of these isolates showed a strong ability to form a biofilm **(Fig. ****S1****).** Our results agree with the previously published study that showed that approximately 50% of *E. faecalis* isolated from raw milk and cheese samples were potent biofilm producers **(**Gajewska et al. 2023). The adhesion of the bacterial cells to the equipment surfaces in the dairy industry enhances the opportunity of these bacterial cells to form biofilm and consequently adversely influences the final product (Srey et al. 2013**;** Kagkli et al. 2007). The resistance of Enterococci is significantly increased by biofilm formation, with consequent impacts on the food industry **(**Abebe 2020**).** Biofilm formation may be affected by the presence or absence of some virulent genes. Our study showed a good correlation between the presence of virulence genes and the ability of *E. faecalis* to form biofilms **(Table S2)** as reported before **(**Cui et al. 2020). A better explanation for this phenomenon may be attributed to the ability of *E. faecalis* with virulence factors to promote bacterial-host adhesion, increase bacterial invasion, and establish biofilms (Creti et al. 2004; Hendrickx et al. 2009).

Enterococcal resistance to antibiotics can be increased significantly by the formation of biofilm **(**Ch’ng et al. 2019). Our results showed that *E. faecalis* isolates exhibited resistance to aminoglycoside antibiotics such as kanamycin (75%), and gentamycin (17.85%). These results are similar to the results published by Jaimee and Halami (2016**)** who reported high resistance of enterococci to aminoglycosides. The resistance of *E. faecalis* to aminoglycoside antibiotics may be due to the reduced uptake of the aminoglycoside and its inability to enter the cell due to the cell permeability reduction or the lack of cytochrome-mediated transport **(**Hollenbeck and Rice 2012**).** We did not observe any resistance to penicillin, ampicillin, and vancomycin (Table S3). Our results agree with many other previous studies that reported that *E. faecalis* isolates were susceptible to chloramphenicol, β-lactams (ampicillin and penicillin), vancomycin, linezolid, and trimethoprim antibiotics **(**Fernández-Fuentes et al. 2014; Kürekci et al. 2016; Chajêcka-Wierzchowska et al. 2019; Sirichoat et al. 2020). Enterococci can acquire resistance to penicillin by a mutation of penicillin-binding proteins (PBPs) or by the production of ß-lactamase, however, they are intrinsically resistant to some β-lactam antibiotics, such as carbapenems and cephalosporins, due to the lack of appropriate PBPs in their structure **(**Zapun et al. 2008; Hollenbeck and Rice 2012; Kraszewska et al. 2022). Considering the intermediate resistance of *E. faecalis* isolates to penicillin and vancomycin, the maximum antibiotic concentrations are required for achieving successful treatment **(**Werner et al. 2008, Hombach et al. 2013).

Our isolated phage vB_EfKS5 against *E. faecalis* showed broad lytic activity against *Enterococcus* spp., and its host range is wider than that reported for other *E. faecalis* phages isolated before. Remarkably, the phage IME-EF1, which shows similarity to the phage vB_EfKS5, infected only 30% (3/10) of *E. faecalis* isolates and 10% (1/10) of *E. faecium* isolates **(**Zhang et al. 2013), whereas phage vB_EfKS5 infected 75.85% (22/29) of *E. faecalis* and 42.85% (3/7) of *E. faecium***(**Table 1**).** On the other hand, phage vB_EfaS_HEf13 showed a broad-spectrum by infecting 70.58% (12/17) of *E. faecalis* isolates but failed to infect *E. faecium* isolates **(**Lee et al. 2019). Broad-host-range phages are highly desirable and could be used in different applications because they are much more efficient and effective against multidrug-resistant bacteria than narrow-host-range phages **(**Khan Mirzaei and Nilsson 2015**).** The isolated phage showed high reproducibility and infectivity, as 70% of *E. faecalis* isolates had an EOP of 0.5 or higher **(Table S4).** Moreover, the one-step growth curve **(**Fig. 2**)** showed that the phage vB_EfKS5 had a short latent period (20 min) and large burst size (183.33 PFU/CFU). Phages with a large burst size are desirable and are considered more virulent as they can effectively eliminate bacterial infections more rapidly **(**Hyman and Abedon 2010**).** Like other previous studies (Chen et al. 2016; Rahmat Ullah et al. 2017; Lee et al. 2019), phage vB_EfKS5 was stable over a wide range of pH values (3–12) and was highly thermostable with residual activity detectable after 30 min exposure at 80 ºC **(**Fig. 2a**).** The stability of phage vB_EfKS5 over a wide temperature and pH range is favorable for its food application under different conditions **(**Fig. 2b**).**

One of the major threats facing the food industry is the biofilms formation by microorganisms present in foods. The biofilm formation on the surfaces of equipment may lead to food contamination and spoilage (Van Houdt and Michiels 2010**).** Bacteriophages and their bacterial hosts develop a significant relationship by modulating the microbial populations (Kiani et al. 2020). Phages can infect their host specifically without disturbing another beneficial microbiota. Consequently, phage could be used as a powerful precise tool to get rid of undesirable bacteria and minimize toxic compounds in foods without having any side effects (del Rio et al. 2019). Phage vB_EfKS5 significantly reduced *E. faecalis* biofilms when they were challenged with different phage MOIs in a microtiter plate assay **(**Fig. 3**).** Tailed phages can penetrate the biofilm matrix and cause lysis of the bacterial cells in the deeper layer. This may be due to the depolymerase enzymatic activity in their tail spike proteins or to the action of endolysin **(**Dakheel et al. 2022).

Genomic analysis revealed that phage vB_EfKS5 belongs to the *Siphoviradae* family, and had a circular, double-stranded DNA genome of 59,246 bp containing 125 open reading frames **(**Table 2**and** Fig. 5**).** The isolated phage vB_EfKS5 shared homology to *Enterococcus* phages such as *Enterococcus* phage vB_EfaS_TV16 (GenBank accession number, MN939408.1) with a coverage of 90%, *Enterococcus* phage vB_OCPT_PG2 (ON113177.1) with a coverage of 89%, *Enterococcus* phage vB_EfaS_HEf13 (MH618488.1) with a coverage of 90%, *Enterococcus* phage VD13 (NC_041861.1) with a coverage of 79%, and *Enterococcus* phage IME-EF1 (NC_041959.1) with a coverage of 89%. Phage vB_EfKS5 has the longest genome (59,246 bp) among all these phages and many unknown genes **(**Table 3**).** These results in addition to the coverage rates suggest that phage vB_EfKS5 might be a novel phage. Importantly, ORF 87 encodes an endolysin protein, a phage enzyme involved in cleaving peptidoglycan bonds in the host cell wall and degrading the murine layer, resulting in the release of new virions **(**Oliveira et al. 2013). The endolysin encoded by phage vB_EfKS5 phage shares a high similarity to the endolysin of phage IME-EF1 (98% identity). The endolysin of phage IME-EF1 had a wider spectrum than the parental phage and rescued the mice infected with a lethal *E. faecalis* with a survival rate of 80% **(**Zhang et al. 2013). Further study of the phage vB_EfKS5 endolysin may promote the future application of these enzymes to control food-borne pathogenic enterococci and boost safety. Importantly, the genome of phage vB_EfKS5 does not encode any lysogenic genes, such as integrases and lytic repressor proteins, indicating that this phage is a lytic phage. Furthermore, there are no ORFs that encode proteins that function as human virulence factors. Our results, therefore, suggest that the phage vB_EfKS5 can be safely used to control the growth of *E. faecalis* in food or medical applications.

A previous study showed the effectiveness of *E. faecalis* phage 156 in reducing tyramine and putrescine final concentration in a designated experiential cheese model **(**del Rio et al. 2019). In this study, we challenged *E. faecalis* by phage vB_EfKS5 alone or in combination with nisin, which is considered a food preservative, to inhibit *E. faecalis* growth in broth and milk. Previously, our lab targeted *S. aureus* isolated from food samples with phage SA46-CTH2 or/ and nisin, and the combination was efficient in controlling the growth of *S. aureus***(**Duc et al. 2020). To our knowledge, this is the first study that uses phage and nisin in combination against *E. faecalis* in food. In agreement with other previous studies (Martínez et al. 2008; Rodríguez-Rubio et al. 2015; Figueiredo and Almeida 2017; Duc et al. 2020), a strong synergy was observed between phage vB_EfKS5 and nisin and the combination was found to be more effective than either treatment alone in reducing *E. faecalis* numbers in broth **(**Fig. 6a**)** and milk **(**Fig. 6b**).** Among those treatments, bacteriophage vB_EfKS5 was very potent and effective in reducing *E. faecalis* growth by 7 log_10_ CFU/ml after 6 h of incubation. The mechanism of phage and nisin to inhibit the *E. faecalis* growth may be attributed to phage lytic enzymes present on phage tail fibers or released during bacterial host lysis which may act synergistically with nisin to destroy bacteria cell wall. Another mechanism may be due to the formation of the pores in the cytoplasmic membrane of the host by the antibacterial action of nisin which subsequently facilitates the phage penetration, infection, and the release of phage progenies **(**Duc et al. 2020). Furthermore, the emergence of phage-resistant bacteria may be suppressed by nisin and decrease the bacterial inoculum, which indirectly increases phage MOI, subsequently enhancing phage efficacy, and vice versa (Martínez et al. 2008). These results indicate that phage vB_EfKS5 is highly effective in inactivating and inhibiting the growth of *E. faecalis*. This data indicates that phage vB_EfKS5 is an enterococcal phage with a broad host range and high productivity and accordingly can be useful to control *Enterococcus* spp. growth in food and medical devices. In future studies, endolysin of phage vB_EfKS5 may be extracted and purified for useful applications in food as a food preservative. In addition, this phage may be used in cocktails with other phages to broaden the host range and extend the shelf-life of food.

### Electronic supplementary material

Below is the link to the electronic supplementary material.

Supplementary Material 1

## Data Availability

All data produced or analyzed during this study are included in this published article and its supplementary information files. The genomic DNA of the phage will be available in the NCBI database when this study is published.
