# Supplementary material for: Potential application of phage vB_EfKS5 to control Enterococcus faecalis and its biofilm in food
Source: AMB Express. 2023 Nov 20;13:130. doi: 10.1186/s13568-023-01628-6 (PMC10661674; doi:10.1186/s13568-023-01628-6)
Supplement: Supplementary file 1 — Supplementary Material 1 [file 13568_2023_1628_MOESM1_ESM.docx]

**Supplementary materials**

**Journal: AMB express**

**Title:** **Potential application of phage vB_EfKS5 to control *Enterococcus faecalis* and its biofilm in food**

Mohamed El-Telbany^1,2†^, Chen-Yu Lin^1†^, Marwa Nabil Abdelaziz^1^, Aye Thida Maung^1^, Ayman El-Shibiny^3^, Tahir Noor Mohammadi^1,4^, Mahmoud Zayda^1,5^, Chen Wang^1^, Su Zar Chi Lwin^1^, Junxin Zhao^1^, Yoshimitsu Masuda^6^, Ken-ichi Honjoh^6*^ and Takahisa Miyamoto^6^

^1^Department of Bioscience and Biotechnology, Graduate School of Bioresource and Bioenvironmental Sciences, Kyushu University

^2^Department of Microbiology and Botany, Faculty of Science, Zagazig University, Zagazig 44519, Egypt

^3^Center for Microbiology and Phage Therapy, Zewail City of Science and Technology, 6th of October City 12578, Egypt

^4^Teagasc Food Research Center, Moorepark, Fermoy, Cork, Ireland.

^5^Department of Food Hygiene and Control, Faculty of Veterinary Medicine, University of Sadat City, Sadat City, Monofiya Governorate, Egypt

^6^Department of Bioscience and Biotechnology, Faculty of Agriculture, Graduate School, Kyushu University, 744 Motooka, Nishi-ku, Fukuoka, 819-0395, Japan

†Co-first Authors: Mohamed El-Telbany and Chen-Yu Lin contributed equally to this manuscript.

***Corresponding author:** Ken-ichi Honjoh, Department of Bioscience and Biotechnology, Faculty of Agriculture, Graduate School, Kyushu University, 744 Motooka, Nishi-ku, Fukuoka, 819-0395, Japan, Tel./Fax.: +81-92-802-4758, email: [honjoh@agr.kyushu-u.ac.jp](mailto:honjoh@agr.kyushu-u.ac.jp)

**Figure S1**


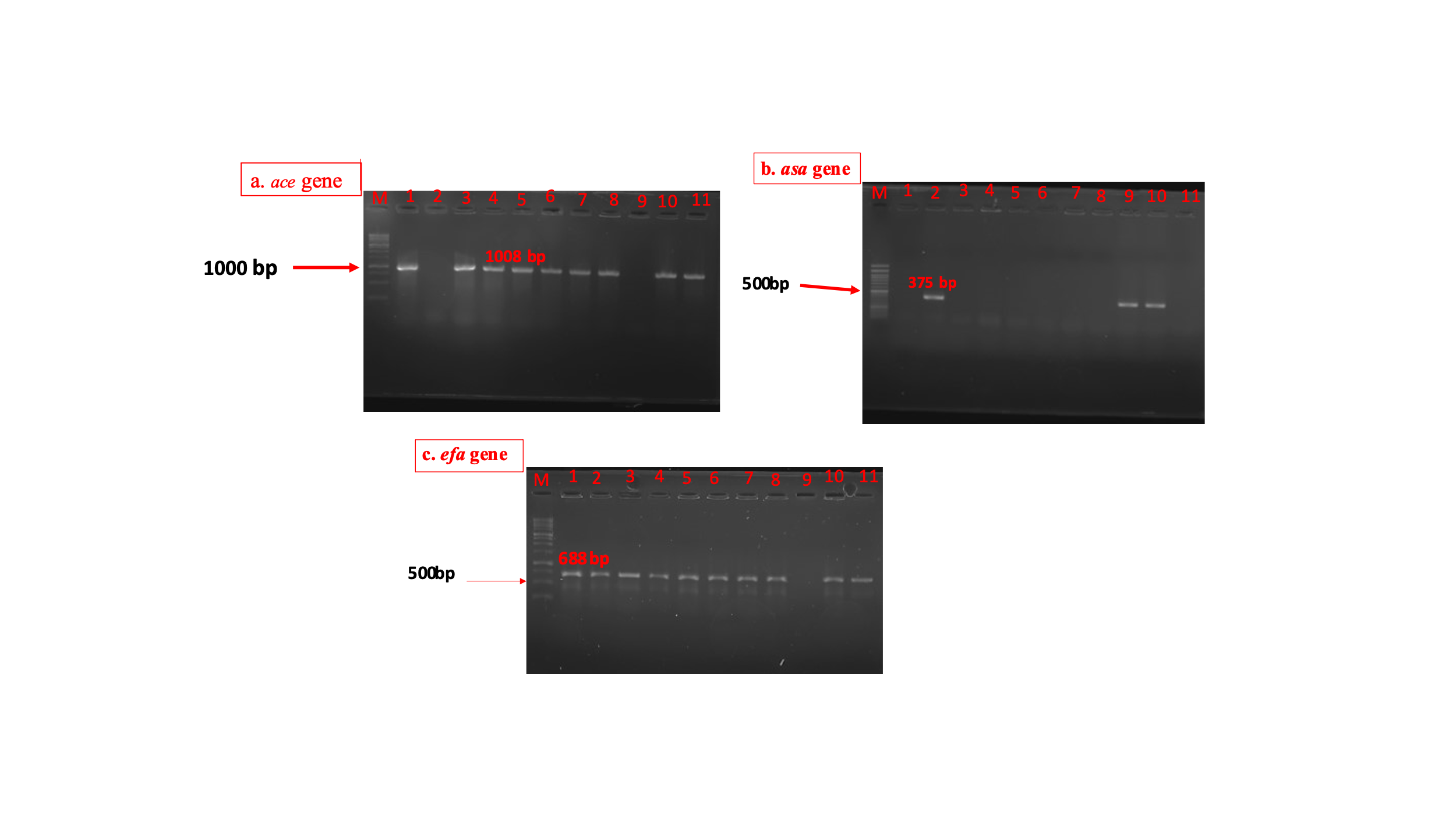


**Figure S2**

**Figure S3**

**Figure S4**

A

**
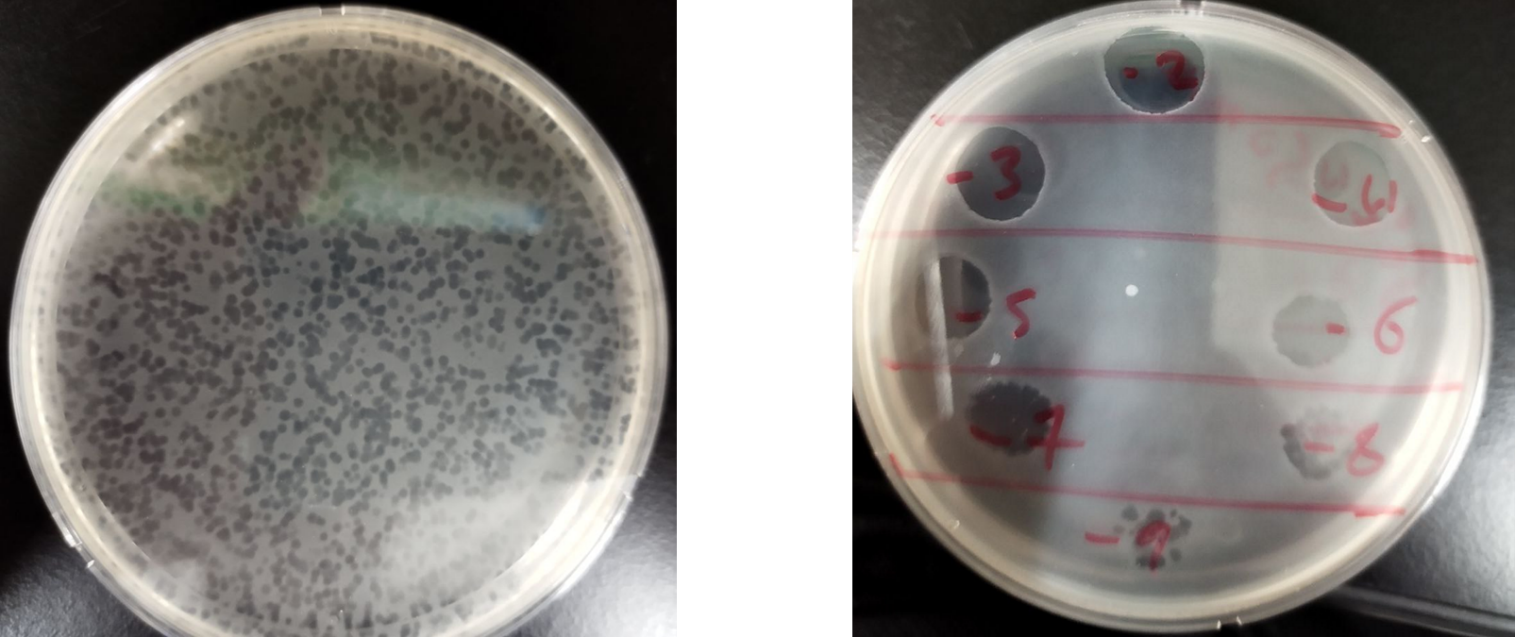
**

**10^-10^**

**10^-9^**

**10^-8^**

**10^-7^**

**10^-6^**

**10^-5^**

**10^-5^**

**10^-3^**

**10^-2^**

B

**Figure S5**


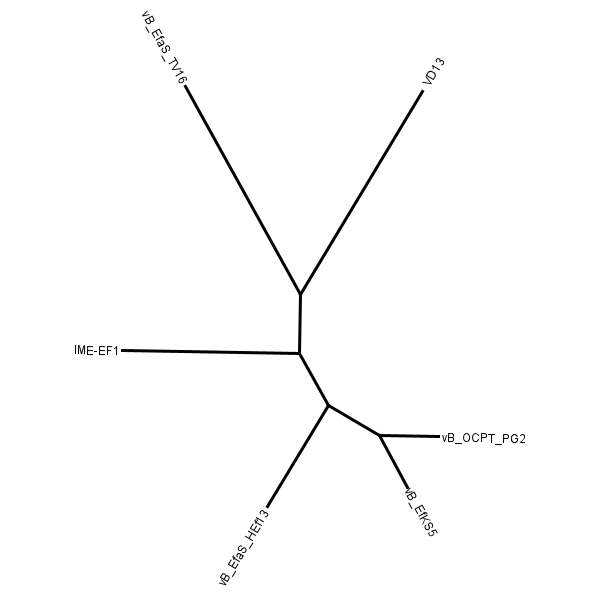


**Figure legends**

**Fig. S1: A gel electrophoresis showing the distribution of virulence genes (*ace*, *asa*, and *efa*) among *E. faecalis*. Lane M: Molecular Marker; Lane 1–11 *E. faecalis* isolates.**

**Fig. S2: Susceptibility pattern of *E. faecalis* isolates against tested antibiotics. Abbreviations as follow: penicillin, EM: Erythromycin, GEN: Gentamycin, KM: kanamycin, RA: Rifampin, VCM: Vancomycin, AMP: Ampicillin, ACV: Amoxicillin- clavulanic acid, and CIP: Ciprofloxacin.**

**Fig. S3: Biofilm formation absorbance by *E. faecalis* isolates. Results are expressed as Mean ±SD from three independent experiments.**

**Fig. S4: Spot test and double agar overlay plaque assays. Graphical representation of the different layers deposited in a Petri dish for (A) a double agar overlay plaque assay and (B) a spot test.**

**Fig. S5: Phylogenetic tree of phage vB_EfKS5 with other *E. faecalis* phages made with the whole genome-based analysis using Geneious prime.**

**Table S1** **Oligonucleotide primers sequences used in PCR**

| Gene | Primer Sequence  5'-3' | Amplified product  (bp) | Reference |
| --- | --- | --- | --- |
| *Asa1* | GCACGCTATTACGAACTATGA | 375 | **Vankerckhoven et al**, 2004 |
|  | TAAGAAAGAACATCACCACGA |  |  |
| ace | GAATTGAGCAAAAGTTCAATCG | 1008 | **Shankar et al.** 1999 |
|  | GTCTGTCTTTTCACTTGTTTC |  |  |
| efaA | GCCAATTGGGACAGACCCTC | 688 | **Creti et al.,** 2004 |
|  | CGCCTTCTGTTCCTTCTTTGGC |  |  |

**Table S2 Prevalence of virulence genes among isolated *E. faecalis* strains**

| ***E. faecalis* isolates** | **Virulence genes** | | |
| --- | --- | --- | --- |
|  | ***Asa1*** | ***ace*** | ***efaA*** |
| ***E. faecalis* 1** | **-** | **+** | **+** |
| ***E. faecalis* 2** | **+** | **-** | **+** |
| ***E. faecalis* 3** | **-** | **+** | **+** |
| ***E. faecalis* 4** | **-** | **+** | **+** |
| ***E. faecalis* 5** | **-** | **+** | **+** |
| ***E. faecalis* 6** | **-** | **+** | **+** |
| ***E. faecalis* 7** | **-** | **+** | **+** |
| ***E. faecalis* 8** | **-** | **+** | **+** |
| ***E. faecalis* 9** | **+** | **-** | **-** |
| ***E. faecalis* 10** | **+** | **+** | **+** |
| ***E. faecalis* 12** | **-** | **+** | **+** |
| ***E. faecalis* 13** | **-** | **-** | **+** |
| ***E. faecalis* 14** | **-** | **+** | **+** |
| ***E. faecalis* 15** | **+** | **+** | **+** |
| ***E. faecalis* 16** | **-** | **-** | **+** |
| ***E. faecalis* 17** | **+** | **+** | **+** |
| ***E. faecalis* 18** | **-** | **+** | **+** |
| ***E. faecalis* 19** | **-** | **+** | **+** |
| ***E. faecalis* 20** | **-** | **+** | **+** |
| ***E. faecalis* 21** | **-** | **+** | **+** |
| ***E. faecalis* 22** | **-** | **+** | **+** |
| ***E. faecalis* 23** | **-** | **-** | **+** |
| ***E. faecalis* 24** | **-** | **+** | **+** |
| ***E. faecalis* 25** | **-** | **+** | **+** |
| ***E. faecalis* 26** | **-** | **+** | **-** |
| ***E. faecalis* 27** | **-** | **+** | **-** |
| ***E. faecalis* 28** | **+** | **-** | **+** |
| ***E. faecalis* 29** | **-** | **+** | **+** |
| ***E. faecalis* JCM7783** | **+** | **+** | **+** |
| **Percentage %** | **21.42** | **78.57** | **89.28** |

**-: negative; +: positive**

Table S3 Antibiotic sensitivity of *E. faecalis* food isolates

| *E. faecalis* isolates | Antimicrobial agents | | | | | | | | |
| --- | --- | --- | --- | --- | --- | --- | --- | --- | --- |
|  | PC | EM | GM | KM | RA | VCM | ABP | ACV | CIP |
| *E. faecalis* 1 | S | IR | S | S | IR | S | S | S | S |
| *E. faecalis* 2 | S | R | S | S | S | S | S | S | S |
| *E. faecalis* 3 | S | IR | IR | R | R | S | S | S | IR |
| *E. faecalis* 4 | S | R | IR | R | IR | S | S | S | R |
| *E. faecalis* 5 | S | IR | R | R | R | IR | S | S | IR |
| *E. faecalis* 6 | S | IR | IR | R | R | IR | S | S | R |
| *E. faecalis* 7 | S | IR | IR | R | R | S | S | S | IR |
| *E. faecalis* 8 | S | S | IR | R | R | S | S | S | S |
| *E. faecalis* 9 | IR | IR | IR | R | R | S | S | S | IR |
| *E. faecalis* 10 | S | S | IR | S | S | S | S | S | S |
| *E. faecalis* 12 | S | IR | R | S | IR | S | S | S | IR |
| *E. faecalis* 13 | IR | IR | R | R | R | S | S | S | R |
| *E. faecalis* 14 | S | IR | IR | S | R | S | S | S | IR |
| *E. faecalis* 15 | S | IR | IR | R | S | S | S | S | S |
| *E. faecalis* 16 | S | IR | IR | R | S | S | S | S | S |
| *E. faecalis* 17 | S | S | IR | R | R | S | S | S | S |
| *E. faecalis* 18 | IR | R | R | R | S | S | S | S | IR |
| *E. faecalis* 19 | S | IR | IR | R | S | S | S | S | IR |
| *E. faecalis* 20 | S | S | IR | R | IR | S | S | S | S |
| *E. faecalis* 21 | S | IR | R | R | S | S | S | S | S |
| *E. faecalis* 22 | S | IR | IR | R | S | S | S | S | R |
| *E. faecalis* 23 | S | IR | IR | R | R | IR | S | S | IR |
| *E. faecalis* 24 | S | S | IR | R | R | S | S | S | R |
| *E. faecalis* 25 | S | IR | IR | IR | R | S | S | S | R |
| *E. faecalis* 26 | S | IR | IR | R | R | S | S | S | R |
| *E. faecalis* 27 | S | IR | S | R | R | IR | S | S | IR |
| *E. faecalis* 28 | S | R | S | R | R | S | S | S | IR |
| *E. faecalis* 29 | S | IR | S | S | S | S | S | S | S |
| Percentage of resistant isolates (%) | - | 14.28 | 17.85 | 75 | 53.57 | - | - | - | 25 |
| Percentage of intermediate resistance isolates (%) | 10.71 | 67.85 | 64.28 | 3.57 | 14.28 | 14.28 | - | - | 39.28 |
| Percentage of sensitive isolates (%) | 89.28 | 17.85 | 17.85 | 21.42 | 32.14 | 85.71 | 100 | 100 | 35.71 |

Abbreviations are as follows: (R): Resistant (S): sensitive (IR): Intermediate resistance. PC: penicillin, EM: Erythromycin, GEN: Gentamycin, KM: kanamycin, RA: Rifampin, VCM: Vancomycin, AMP: Ampicillin, ACV: Amoxicillin- clavulanic acid, and CIP: Ciprofloxacin.

**Table S4 Efficiency of plating (EOP)**

| *E. faecalis* isolates | Average PFU/ml (±SD) | EOP | Production |
| --- | --- | --- | --- |
| *E. faecalis7* (Host*)* | 10.47±0,17 |  |  |
| *E. faecalis 2* | 1.4±0.12 | 0.1 | Medium |
| *E. faecalis 3* | 9±0.2 | 0.85 | High |
| *E. faecalis 4* | 3.47 ±0.23 | 0.33 | Medium |
| *E. faecalis 5* | 9 ±0.25 | 0.85 | High |
| *E. faecalis 6* | 10.3±0.16 | 0.98 | High |
| *E. faecalis 8* | 1.3±0.18 | 0.1 | Medium |
| *E. faecalis 9* | 10.3±0.2 | 0.98 | High |
| *E. faecalis 12* | 10.3±0.2 | 0.98 | High |
| *E. faecalis 13* | 9 ±0.2 | 0.85 | High |
| *E. faecalis 16* | 9.3±0.2 | 0.88 | High |
| *E. faecalis 18* | 5.3±0.36 | 0.5 | High |
| *E. faecalis 19* | 9.44± 0.23 | 0.9 | High |
| *E. faecalis 20* | 1.3±0.25 | 0.1 | Medium |
| *E. faecalis 22* | 1.2±0.23 | 0.1 | Medium |
| *E. faecalis 23* | 10.47±0.18 | 1.00 | High |
| *E. faecalis 25* | 9.3±0.2 | 0.88 | High |
| *E. faecalis 26* | 10.00±0.22 | 0.96 | High |
| *E. faecalis 27* | 7.00±0.1 | 0.67 | High |
| *E. faecalis 28* | 1.2±0.23 | 0.1 | Medium |
| *E. faecalis 29* | 9.42± 0.23 | 0.9 | High |
